# Basal-Like Cell-Conditioned Medium Exerts Anti-Fibrotic Effects *In Vitro* and *In Vivo*

**DOI:** 10.3389/fbioe.2022.844119

**Published:** 2022-03-08

**Authors:** Petra Khan, Kleanthis Fytianos, Sabrina Blumer, Julien Roux, Amiq Gazdhar, Spasenija Savic, Lars Knudsen, Danny Jonigk, Mark P. Kuehnel, Sofia Mykoniati, Michael Tamm, Thomas Geiser, Katrin E. Hostettler

**Affiliations:** ^1^ Department of Biomedicine and Clinics of Respiratory Medicine, University Hospital Basel, University of Basel, Basel, Switzerland; ^2^ Department of Pulmonary Medicine, University Hospital Bern, and Department of Biomedical Research, University of Bern, Bern, Switzerland; ^3^ Swiss Institute of Bioinformatics, Basel, Switzerland; ^4^ Institute of Medical Genetics and Pathology, University Hospital Basel, University of Basel, Basel, Switzerland; ^5^ Institute of Functional and Applied Anatomy, Hannover Medical School, Hannover, Germany; ^6^ Biomedical Research in Endstage and Obstructive Lung Disease Hannover (BREATH), Member of the German Center for Lung Research (DZL), Hannover, Germany; ^7^ Institute of Pathology, Hannover Medical School, Hannover, Germany; ^8^ Department of Internal Medicine, Jura Cantonal Hospital, Delemont, Switzerland

**Keywords:** basal cells, idiopathic pulmonary fibrosis, conditioned medium, PGE2, lung fibroblasts, aberrant basaloid cells

## Abstract

In idiopathic pulmonary fibrosis (IPF), basal-like cells are atypically present in the alveolar region, where they may affect adjacent stromal cells by paracrine mechanisms. We here aimed to confirm the presence of basal-like cells in peripheral IPF lung tissue *in vivo*, to culture and characterize the cells *in vitro,* and to investigate their paracrine effects on IPF fibroblasts *in vitro* and in bleomycin-injured rats *in vivo*. Basal-like cells are mainly localized in areas of pathological bronchiolization or honeycomb cysts in peripheral IPF lung tissue. Single-cell RNA sequencing (scRNA-seq) demonstrated an overall homogeneity, the expression of the basal cell markers cytokeratin KRT5 and KRT17, and close transcriptomic similarities to basal cells in the majority of cells cultured *in vitro*. Basal-like cells secreted significant levels of prostaglandin E2 (PGE2), and their conditioned medium (CM) inhibited alpha-smooth muscle actin (α-SMA) and collagen 1A1 (Col1A1) and upregulated matrix metalloproteinase-1 (MMP-1) and hepatocyte growth factor (HGF) by IPF fibroblasts *in vitro*. The instillation of CM in bleomycin-injured rat lungs resulted in reduced collagen content, improved lung architecture, and reduced α-SMA-positive cells. Our data suggested that basal-like cells may limit aberrant fibroblast activation and differentiation in IPF through paracrine mechanisms.

## Introduction

Idiopathic pulmonary fibrosis (IPF) is a chronic and irreversible interstitial lung disease characterized by a progressive destruction of the lung parenchyma and the respective loss of lung function (Raghu et al., 2011). The initial events leading to the development and progression of IPF are still not elucidated; however, evidence suggests that repeated injury to the alveolar epithelial cells plays a central role (Selman et al., 2001). Typically, alveolar regeneration involves the self-renewal of alveolar epithelial-type (AT2) cells and their differentiation into (AT1) cells (Barkauskas et al., 2013). During normal lung homeostasis, there is no overlap of epithelial cell types of the alveolar and the conducting regions. However, after severe lung injury in mice and humans, an expansion of airway basal-like cells within the alveolar region was observed (Kumar et al., 2011; Vaughan et al., 2015; Zuo et al., 2015; Ray et al., 2016; Xi et al., 2017; Taylor et al., 2018; Yang et al., 2018; Fernanda de Mello Costa et al., 2020). In line with these findings, a decline in alveolar epithelial cells, corresponding to an expansion of cells expressing airway epithelial markers in IPF versus control lung tissue, was demonstrated (Xu et al., 2017; Adams et al., 2020; Habermann et al., 2020). Furthermore, the disease-enriched presence of basal-like cells (Chilosi et al., 2002; Smirnova et al., 2016; Xu et al., 2017; Prasse et al., 2019) and a novel KRT5-/KRT17+ aberrant basaloid cell population (Adams et al., 2020; Habermann et al., 2020) within the alveolar compartment and in bronchoalveolar lavage (BAL) fluid of IPF patients was described. In mice, where basal cells are restricted to the trachea and mainstem bronchi, these alveolar basal-like cells arise from a distinct intrapulmonary p63+ stem/progenitor cell population (Xi et al., 2017; Yang et al., 2018). In humans, the origin of alveolar basal-like cells is less clear: in contrast to mice, basal cells are present throughout the airways in humans (Rock et al., 2010). Therefore, migration of distal airway basal cells into the alveolar region after lung injury in humans is likely (Fernanda de Mello Costa et al., 2020).

While the occurrence of basal-like cells within the alveolar region in injured lungs is a widely accepted phenomenon, their role and function remains largely unknown. It was suggested that basal-like cells may help to regenerate the injured alveolar epithelium by differentiation into AT2 cells in rodent lungs (Kumar et al., 2011; Vaughan et al., 2015; Zuo et al., 2015). On the contrary, the cells’ disease-enriched presence and a report of a correlation between increased mortality and the appearance of basal-like cells in BAL fluid of IPF patients (Prasse et al., 2019) would rather point to a non-beneficial role of these cells in lung fibrosis. In addition to their capacity to differentiate into specialized cells, different types of stem/progenitor cells have been shown to exert their effects through their secretome by paracrine mechanisms (Xia et al., 2019). In this study, we cultured KRT5+/KRT17+ basal-like cells from peripheral fibrotic lung tissue and investigated the effects of their conditioned medium (CM) on cultured primary human IPF fibroblasts and in a rat model of pulmonary fibrosis.

## Materials and Methods

### Cell Culture

Basal-like cells or fibroblasts were cultured from peripheral fibrotic lung tissue of IPF or other interstitial lung disease patients. Tissue was cut into small pieces and placed into 12-well culture plates (one tissue piece/well) containing growth medium (DMEM with 10% FCS, 25 mM HEPES, 1× (vol/vol) sodium pyruvate, 1× (vol/vol) MEM vitamin-mix, and 1× (vol/vol) antibiotic-antimycotic). Tissue pieces, which showed outgrowth of basal-like cells within 2–5 days, proved large numbers of KRT17+/KRT5+ basal-like cells in subsequent IF-staining (Supplementary Figure S1A). Contrariwise, in tissue pieces without outgrowth of basal-like cells but with sprouting of fibroblasts after approximately 7 days, KRT17+/KRT5+ basal-like cells were absent or present in low numbers in subsequent IF-staining (Supplementary Figure 1B).

Both cell types could clearly be differentiated by their different morphologies (Supplementary Figures 2A,B). CM was collected of pure basal-like cell cultures after 5 days. For experiments, fibroblasts were kept in starving medium (DMEM with 0.1% FCS, 25 mM HEPES, 1× (vol/vol) sodium pyruvate, 1× (vol/vol) MEM vitamin-mix, and 1× (vol/vol) antibiotic-antimycotic) for 24 h prior to treatment with the control medium (DMEM with 10% FCS, 25 mM HEPES, 1× (vol/vol) sodium pyruvate, 1× (vol/vol) MEM vitamin-mix, and 1× (vol/vol) antibiotic-antimycotic), basal-like cell-CM, fibroblast (F)-CM, or several different recombinant factors (IL-17A, osteopontin, ENA-78, GM-CSF, HGF, GDF-15, GROα, IL-6, PGE2, or IL-8) ± chemical inhibitors AH6809 (20 μM), PF04418948 (1–10 μM), or indomethacin (1–10 μM). IPF was diagnosed based on ATS/ERS guidelines (Raghu et al., 2011; Raghu et al., 2018). The local ethical committee of the University Hospital, Basel, Switzerland (EKBB05/06), and of the Medical University of Hannover, Germany (2699–2015), approved the culture of human primary lung cells. All materials and instruments used in this study are listed in Supplementary Tables 1, 2.

### Human Lung Tissue Staining

Peripheral human lung tissue was fixed in paraformaldehyde and embedded in paraffin. The paraffin blocks were cut, and routine hematoxylin and eosin (H&E) and immunofluorescence (IF) stainings were performed as described in Supplementary Material.

### TaqMan RT-PCR, Immunoblotting, Immunofluorescence, ELISA, and Cytokine Array

TaqMan^®^ PCR, immunoblotting, and IF stainings were performed as previously described (Hostettler et al., 2017; Seidel et al., 2009; Seidel et al., 2011). Details of primer and antibodies are listed in Supplementary Table S1. Mediators were measured in supernatants by using the DuoSet ELISA development systems, Prostaglandin E2 Parameter Assay Kit, or a Proteome Profiler™ Human XL Cytokine Array kit according to the manufacturers’ instructions. Col1A1 deposition was analyzed by a cell-based ELISA as previously described (Lambers et al., 2014).

### RNA Sequencing

scRNA-seq datasets were generated at the Genomics Facility Basel of the ETH Zurich, Basel, and data were analyzed by the Bioinformatics Core Facility, Department of Biomedicine, University of Basel. The processed scRNA-seq data can be obtained from the Gene Expression Omnibus (GEO) database under accession no. GSE145439. A detailed description of the analysis methods used for these datasets can be found in Supplementary Material.

### Animals

Male Fisher F344 rats (220–240 g) were obtained from Charles River Laboratories GmbH, Sulzfeld, Germany. Experiments were performed in accordance with the standards of the European Convention of Animal Care. The study protocol was approved by the University of Bern Animal Study Committee (BE 135/16).

### Instillation of Bleomycin

On day one of the protocol, F344 rats (220–240 g) were anesthetized by inhalation of 4% isoflurane in a glass chamber, intubated with a 14 GA i.v catheter, and instilled intratracheally with bleomycin (1.28 U/rat) to both lungs.

#### Instillation of Conditioned Media

Seven days after the instillation of bleomycin, rats were anesthetized as described above and divided into five groups with n = 5 in each group (A, B, C, D, and E). Group A received intratracheal administration of control media (DMEM without FBS), group B received fibroblast conditioned media (F-CM), and group C received basal-like cell-CM. Additional groups studied were group D receiving basal-like cell-CM pretreated with prostaglandin (PGE2) neutralizing antibody and group E receiving isotype control antibody (IgG) (n = 3). A volume of 500 µl was administered intratracheally to both the lungs in each group.

#### Assessment

On day 7 after basal-like cell-CM and control media instillation, the animals were anesthetized as described above. Thiopental (50 mg/kg body weight) was administered intraperitoneally. The heart–lung block was explanted, and tissue was collected for further analysis. Routine H&E staining, Sirus red staining, immunohistochemistry, and hydroxyproline assay were performed as described in Supplementary Material.

#### Rat Lung Tissue Homogenization for Single-Cell Isolation and Flow Cytometry

The entire right lung was minced in a 10 cm Petri dish in a sterile solution of RPMI 1640 containing 0.1% collagenase I and 0.25% collagenase II as described previously (Tamo et al., 2018). Cell suspensions were washed, stained, and analyzed as described in detail in Supplementary Material.

#### Data Analysis

All experiments were performed in primary human lung fibroblasts derived from at least three different patients, of which each fibroblast cell line was treated with basal-like cell-CM or F-CM from up to seven different patients. Where applicable, data were expressed as mean ± standard error of the mean (SEM), and Student`s *t*-test was used for statistical comparisons. *p* values ≤ 0.05 were considered as significant.

## Results

### Basal-Like Cells Derived From Peripheral Fibrotic Lung Tissue

**FIGURE 1:**
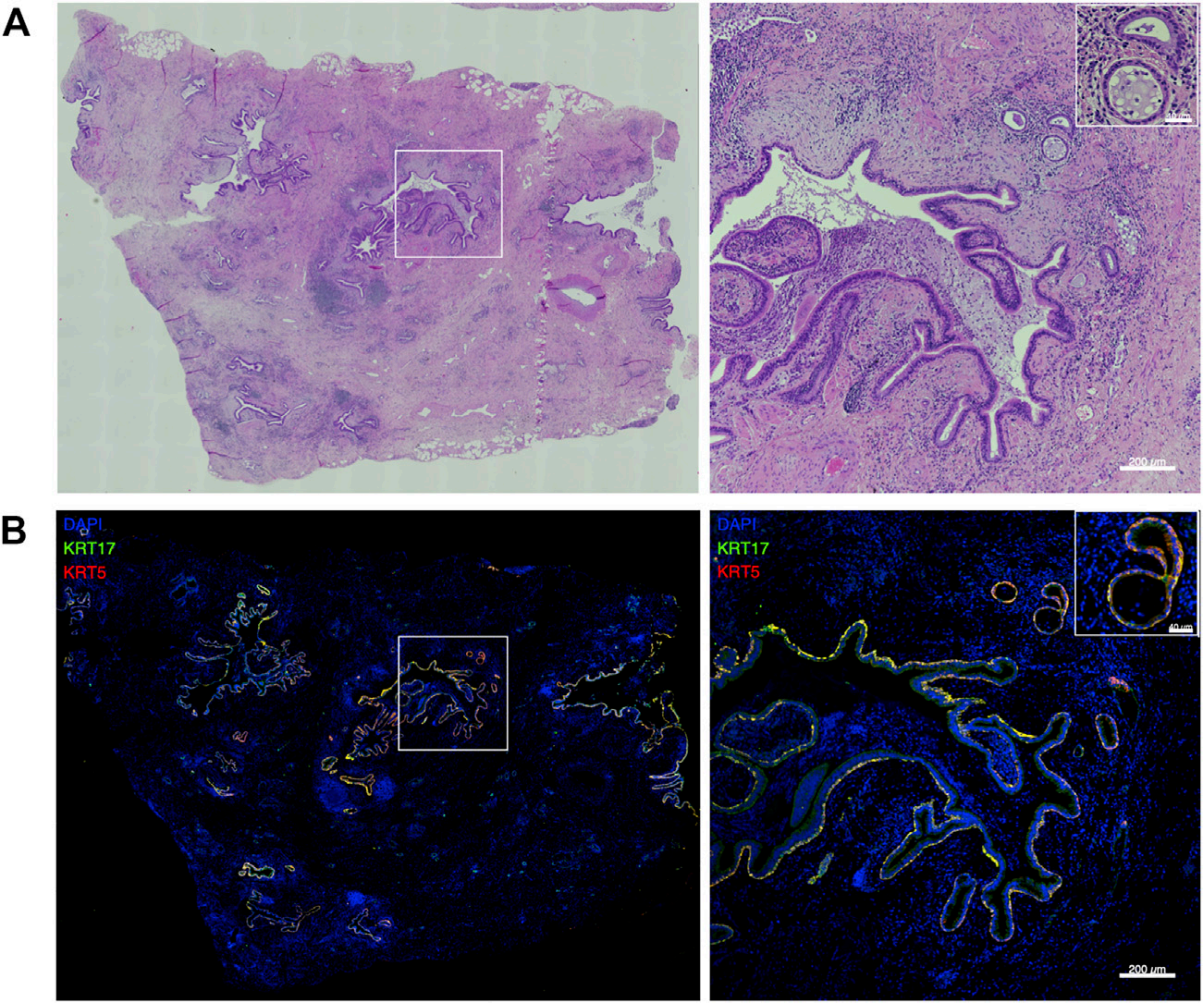
Basal-like cells in peripheral IPF lung tissue. Representative large image composed of stitched ×20 images and the indicated region (white square) at higher magnification showing hematoxylin and eosin (H&E) stainings **(A)** and merged images of KRT5 (red) and KRT17 (green) stainings of peripheral lung tissue of IPF patients (n = 5) **(B)**.

KRT5 and KRT17 positive basal-like cells were detected in peripheral lung tissue of five different IPF patients where they were mainly localized within the areas of pathological bronchiolization and honeycomb cysts (Figures 1A,B).

**FIGURE 2:**
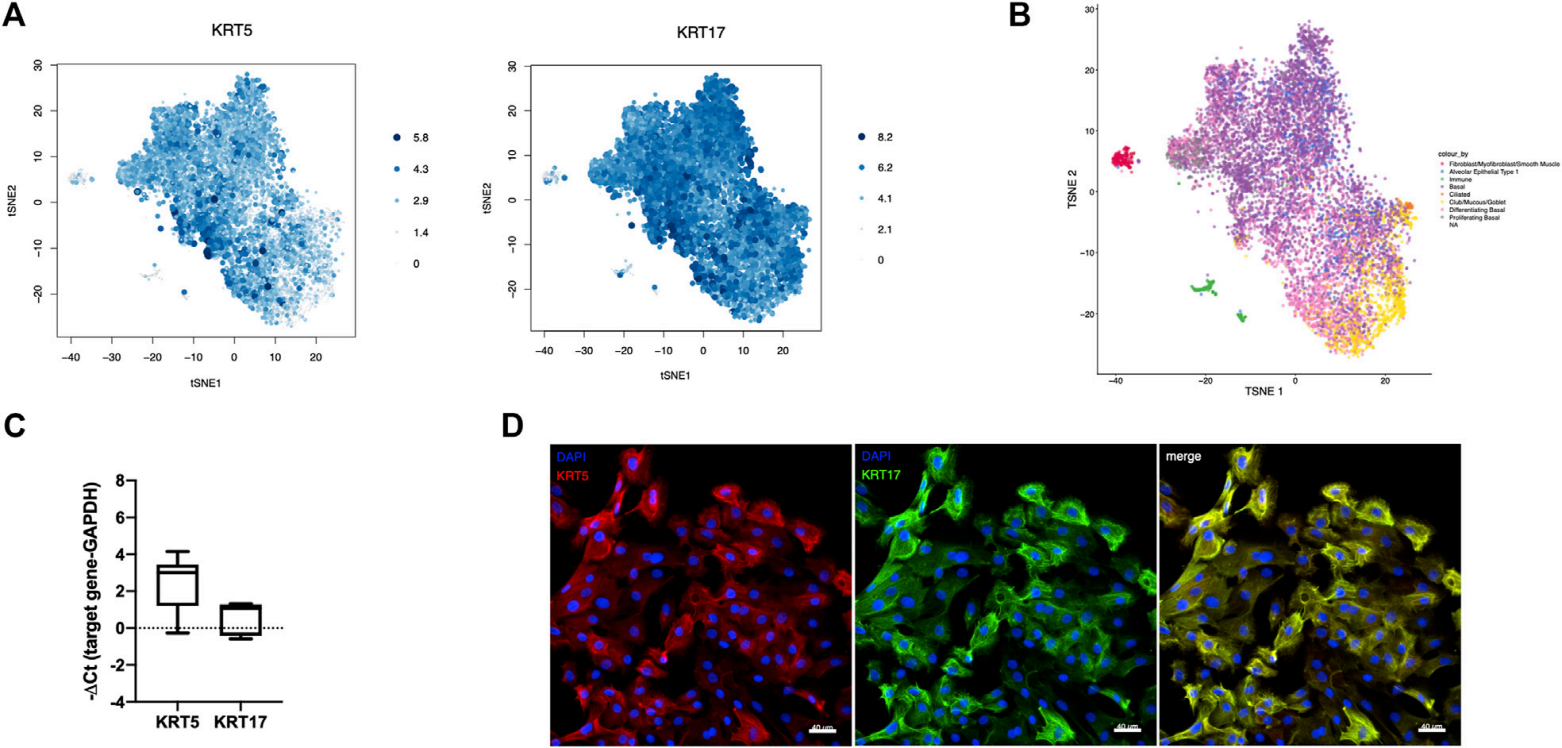
Characteristics of cultured basal-like cells. scRNA-seq was performed in basal-like cells derived from three different IPF patients, and the expression levels of the basal cell markers KRT5 and KRT17, proportional to the size and color intensity of the dots, are shown on a t-SNE plot **(A)**. t-SNE shows the best matching cell type for each cell after annotation of cultured cells using as reference an scRNA-seq atlas of cell types in the human lung **(B)**. KRT5 (n = 11) and KRT17 (n = 5) RNA expression was confirmed by TaqMan PCR **(C)**. Representative images showing staining for KRT5 (red), left, and KRT17 (green), middle, and their merged image, right in cultured basal-like cells of four different IPF patients **(D)**.

After quality control and filtering, a total of 8,953 basal-like cells derived from three different IPF patients were analyzed by scRNA-seq. scRNA-seq. data demonstrated homogeneity of the cell population with the majority of cells expressing the basal cell markers KRT5 (75%) and KRT17 (96%) (Figure 2A). Annotation of cultured cells using as reference an scRNA-seq atlas of cell types in the human lung (Travaglini et al., 2020) revealed the closest transcriptomic similarity to basal cells (basal, differentiating basal, and proliferating basal) of the majority of cells (79.9%) (Figure 2B). From the remaining cells, a small percentage of cells matched best to mesenchymal (fibroblasts/myofibroblasts/smooth muscle) (2.0%), AT cell type 1 (5.7%), immune (2.2%), ciliated (0.4%), or secretory (club/mucous/goblet) (10%) epithelial cells (Figure 2B). High expression of KRT5 and KRT17 was confirmed in 5–11 additional patients by TaqMan PCR (Figure 2C) and on the protein level by IF in cells derived from four different patients (Figure 2D).

### Anti-Fibrotic Effects of Basal-Like Cell-CM in Cultured IPF Fibroblasts

**FIGURE 3:**
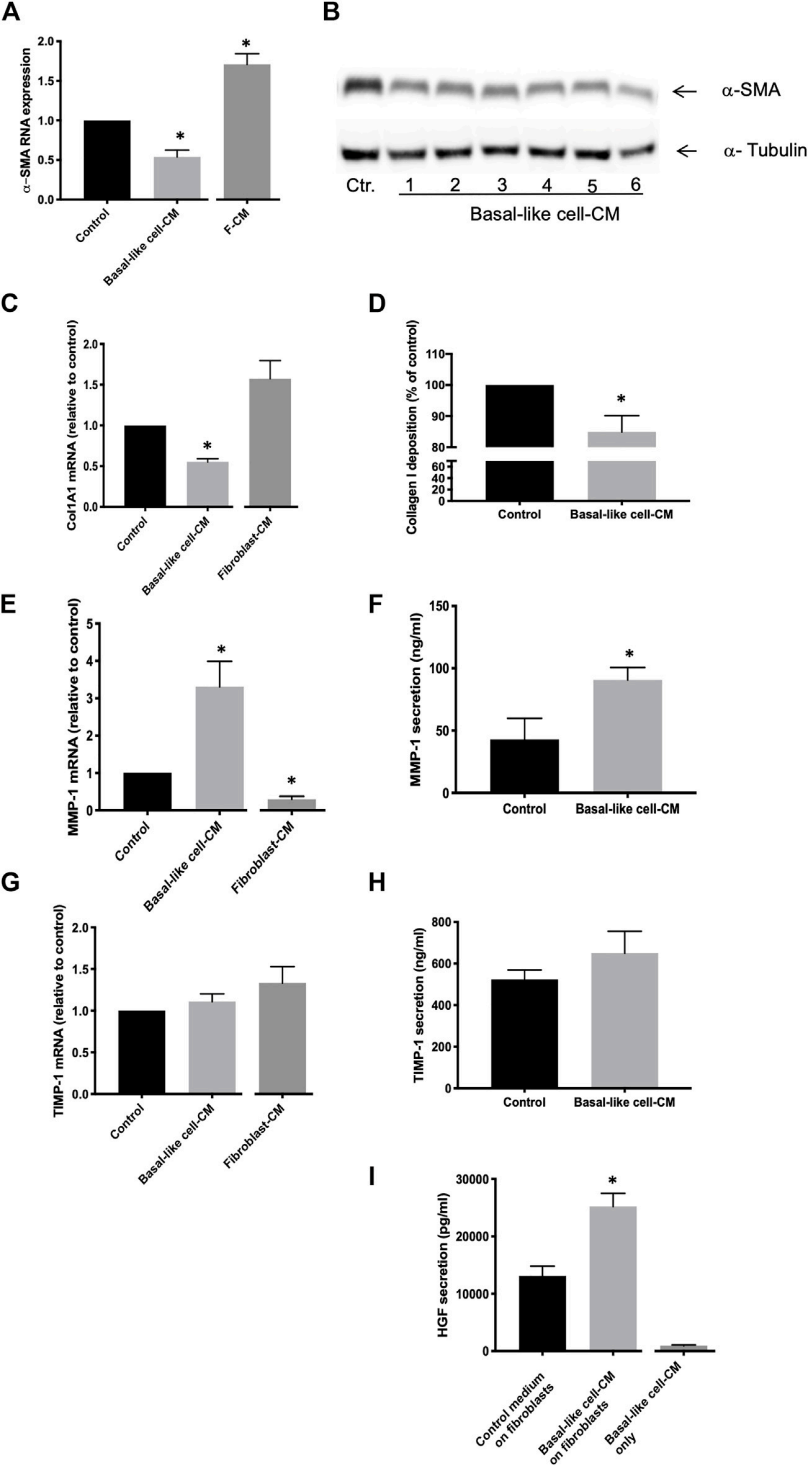
Anti-fibrotic effects of basal-like cell-CM on fibroblasts *in vitro*. Fibroblasts were treated with the control medium, basal-like cell-conditioned medium (CM), or fibroblasts-CM (F-CM). α-SMA RNA expression was measured by TaqMan RT-PCR (n = 3) **(A)**, and changes in α-SMA protein in fibroblasts (n = 3) treated with basal-like cell-CM of six different patients (1–6) were determined by immunoblotting **(B)**. Col1A1 RNA expression was determined after 24 h (n = 7) **(C)** and Col1A1 deposition after 48 h (n = 5) **(D)**. MMP-1 or TIMP-1 RNA expression **(E,G)** or protein secretion **(F,H)** was measured after 24 h. Data are expressed as fold change or as a percentage of fibroblasts treated with control medium (RNA) or in ng/ml (protein). HGF in supernatants of fibroblasts (n = 12) treated with basal-like cell-CM, control medium, or in basal-like cell-CM before the addition to fibroblasts (basal-like cell-CM only) **(I)**.*p < 0.05.

The α-SMA RNA expression of IPF fibroblasts (n = 3) in the control medium was significantly downregulated after incubation with basal-like cell-CM and upregulated in fibroblasts incubated with F-CM (Figure 3A). α-SMA protein expression in IPF fibroblasts (n = 3) was reduced by basal-like cell-CM derived from six different patients (1–6; Figure 3B). α-Tubulin served as a control for equal protein loading.

Col1A1 RNA expression in IPF fibroblasts (n = 7) was significantly inhibited in basal-like cell-CM treated cells when compared to those in the control medium (Figure 3C). Similarly, collagen I deposition in IPF fibroblasts (n = 5) was significantly reduced by 15% ± 2.38 after treatment with basal-like cell-CM (Figure 3D). In contrast, when fibroblasts (n = 3) were treated with F-CM, Col1A1 RNA expression was upregulated in F-CM-treated cells when compared to the medium control (Figure 3C).

MMP-1 RNA expression in IPF fibroblasts (n = 6) was significantly increased by treatment with basal-like cell-CM and reduced by treating the cells with F-CM (Figure 3E). MMP-1 secretion was increased from 42.98 ± 16.99 ng/ml in the control medium to 90.74 ± 9.81 ng/ml in basal-like cell-CM-treated fibroblasts (Figure 3F). Basal-like cell-CM or F-CM had no significant effect on TIMP-1 RNA expression or protein secretion (Figures 3G,H). Basal-like cell-CM medium upregulated the secretion of anti-fibrotic HGF by IPF fibroblasts (Figure 3I): IPF fibroblasts (n = 12) in the control medium secreted HGF at a concentration of 13,125 ± 1,679 pg/ml, which was significantly increased to 25,205 ± 2,302 pg/ml HGF after the addition of basal-like cell-CM for 24 h. Basal-like cell-CM only (before addition to fibroblasts) contained 946.7 ± 114 pg/ml HGF (Figure 3I).

### Mediators Secreted by Basal-Like Cells and Their Effects on IPF Fibroblasts

**FIGURE 4:**
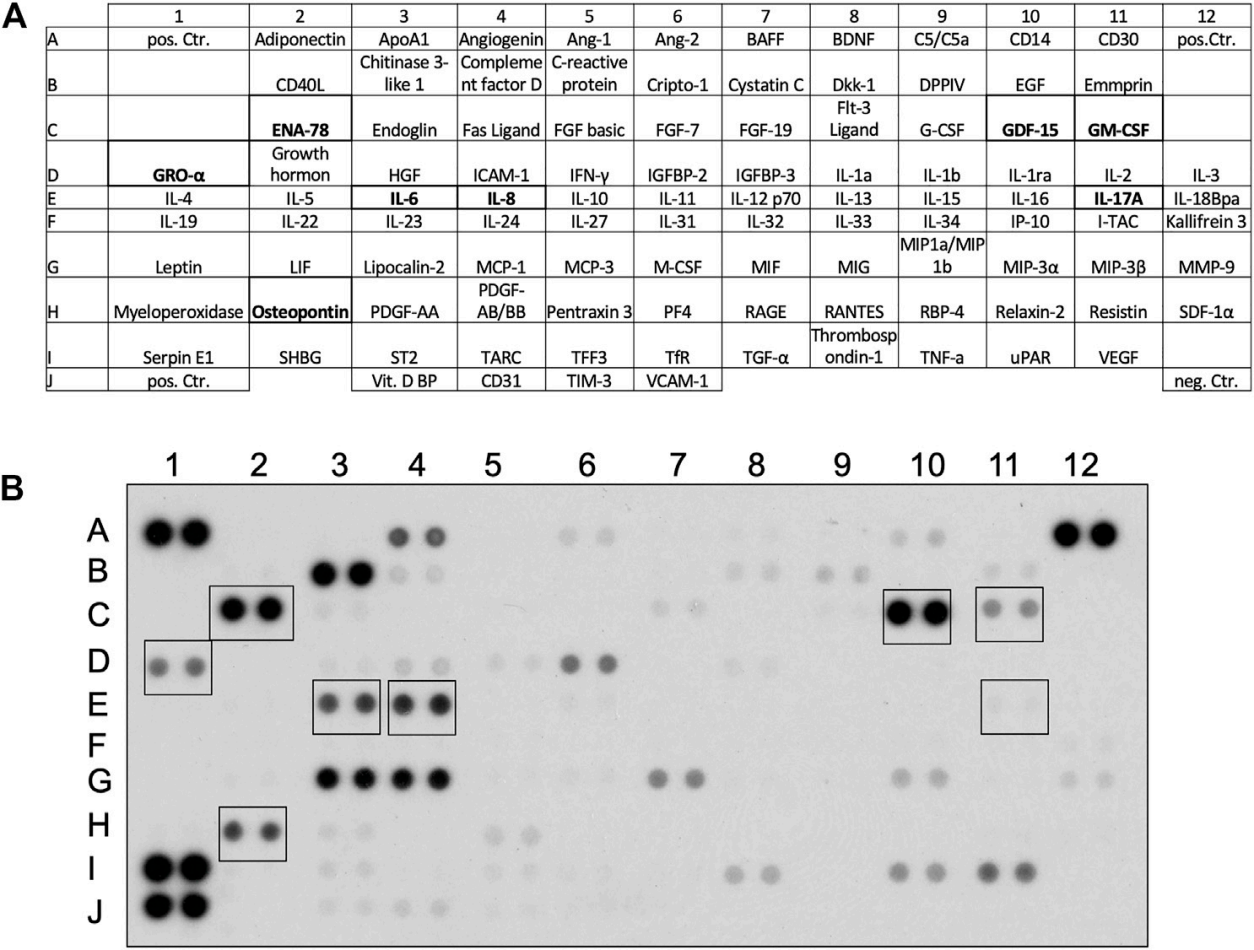
Cytokines detected in the basal-like cell-CM. Proteome Profiler™ Human XL Cytokine Arrays were incubated with basal-like cell-CM derived from three different patients. Detectable cytokines and their location on the array **(A)**. Cytokines highlighted in **(A)** and framed in a representative cytokine array **(B)** were selected for further experiments.

Cytokine arrays were incubated with basal-like cell-CM (n = 3, Figure 4). Several cytokines were detected, of which eight [Figure 4, framed in the cytokine array (B) and highlighted in the table (A)] were selected to test their effects on Col1A1, MMP-1, TIMP-1, or HGF by fibroblasts. In addition, PGE2 was measured by ELISA: In basal-like cell-CM of 36 different patients, an average concentration of 3,034 ± 228.4 pg/ml PGE2 was detected.

**FIGURE 5:**
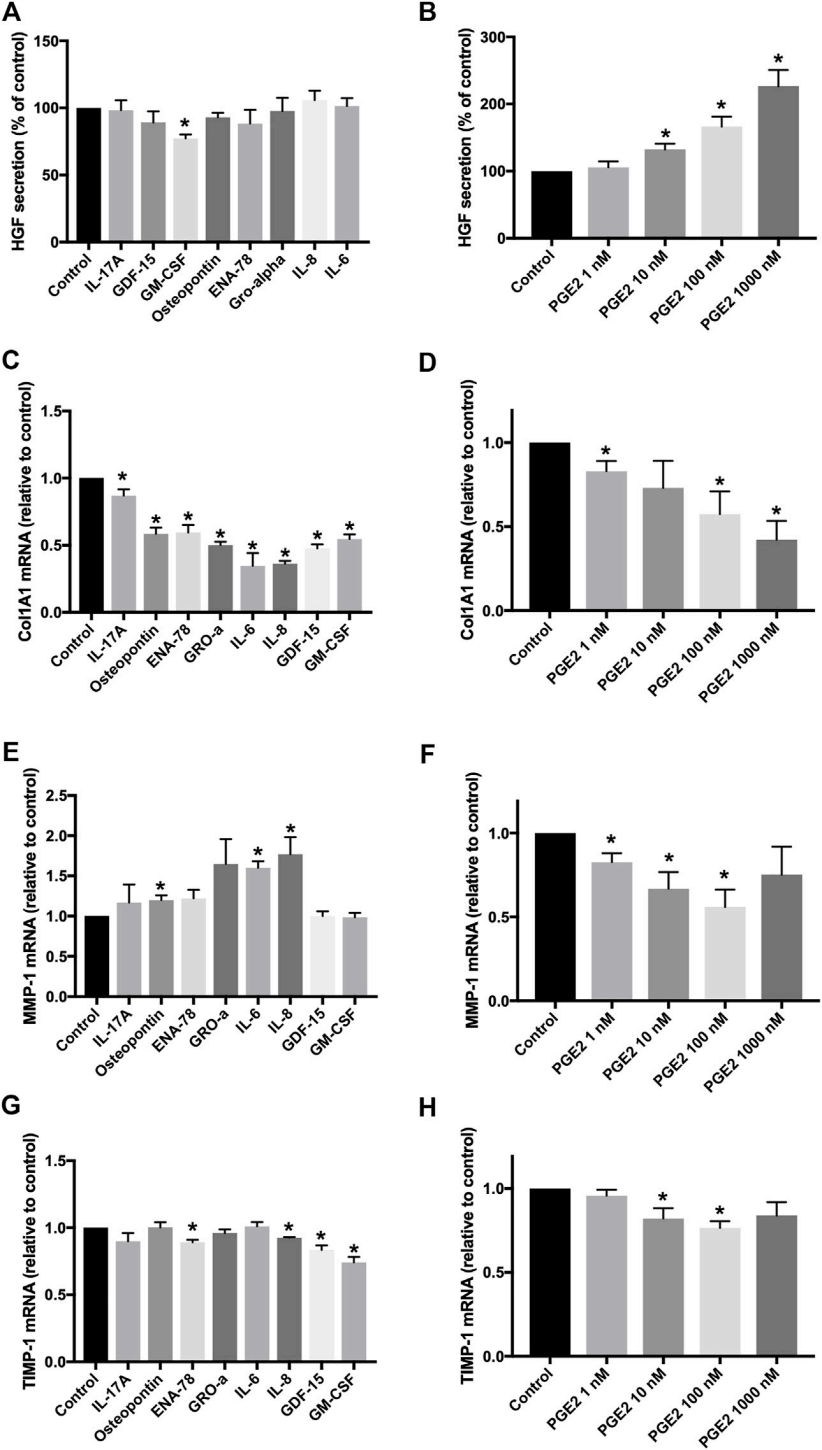
Effects of different recombinant cytokines and PGE2 on fibroblasts *in vitro*. Fibroblasts were treated with eight different recombinant human cytokines (all at 10 ng/ml) or with PGE2 (1–1,000 nM) for 24 h. HGF secretion was measured in cell supernatants, and data were expressed as a percentage of fibroblasts treated with the control medium **(A,B)**. Col1A1 **(C,D)**, MMP-1 **(E,F),** and TIMP-1 **(G,H)** RNA expression was determined by TaqMan RT-PCR, and values were expressed as fold change of fibroblasts treated with the control medium. *p < 0.05.

IPF fibroblasts were treated with eight human recombinant cytokines (all at 10 ng/ml), IL-17A, osteopontin, ENA-78, GM-CSF, GDF-15, GRO-α, IL-6, and IL-8, or with PGE2 (1–1,000 nM). None of the eight tested cytokines upregulated the secretion of HGF by IPF fibroblasts (Figure 5A). GM-CSF weakly but significantly inhibited HGF secretion when compared to the medium control (Figure 5A). Only PGE2 increased HGF secretion by IPF fibroblasts in a dose-dependent manner between 10–1,000 nM PGE2 (Figure 5B). Col1A1 expression was significantly reduced by all tested cytokines (Figure 5C) and by PGE2 (Figure 5D). MMP-1 was significantly increased by osteopontin, IL-6, or IL-8 (Figure 5E), whereas PGE2 significantly downregulated MMP-1 expression (Figure 5F). TIMP-1 expression was significantly downregulated by ENA-78, IL-8, GDF-15, GM-CSF (Figure 5G), or PGE2 at 10–100 nM (Figure 5H).

### Basal-Like Cell-CM Up-Regulates HGF *via* PGE2 in IPF Fibroblasts

**FIGURE 6:**
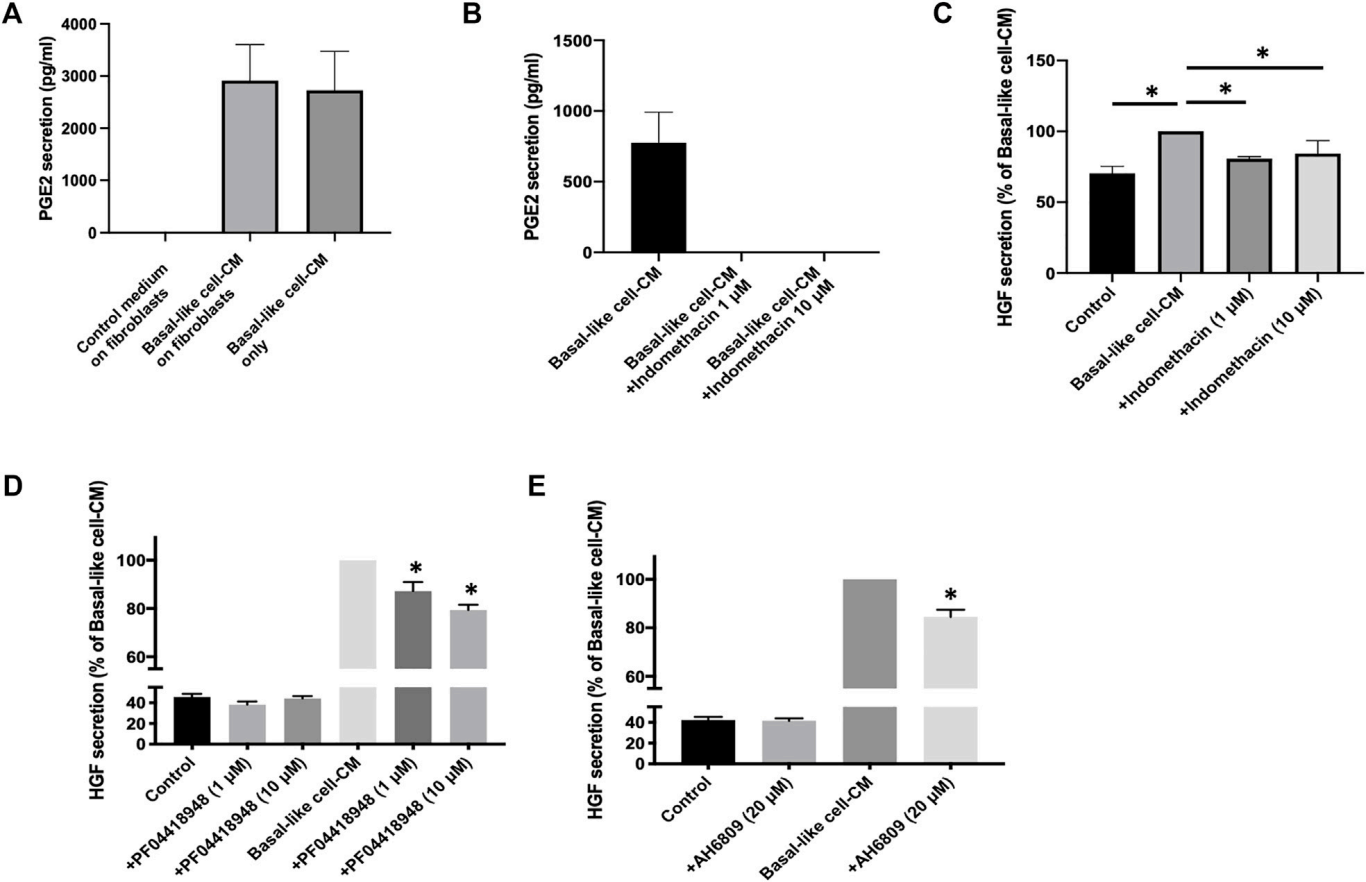
PGE2 mediates basal-like cell-CM-induced HGF in IPF fibroblasts. PGE2 in supernatants of fibroblasts (n = 6) treated with basal-like cell-CM, control medium, or basal-like cell-CM before the addition to fibroblasts **(A)**. PGE2 secretion from basal-like cells with and without COX inhibitor indomethacin (1 and 10 μM) **(B)**. Data are expressed as pg/ml. IPF fibroblasts (n = 4) were incubated with the control medium or basal-like cell-CM collected from basal-like cells treated with or without indomethacin (1 and 10 μM). After 24 h, HGF secretion was measured in the cell supernatants, and data were expressed as a percentage of fibroblast HGF secretion treated with basal-like cell-CM alone **(C)**. IPF fibroblasts (n = 4) were treated with the control medium or basal-like cell-CM with or without PGE2 receptor antagonists PF04418948 [**(D)**, 1 and 10 μM] or AH6809 [**(E)**, 20 μM]. HGF secretion was measured, and values are expressed as a percentage of fibroblast HGF secretion treated with basal-like cell-CM **(D, E)**. *p < 0.05.

Our data indicate that basal-like cell-CM-induced HGF secretion by fibroblasts is mainly regulated by PGE2. When fibroblasts (n = 6) were incubated with either the control medium or basal-like cell-CM, PGE2 levels in the control medium were below the detection limit (Figure 6A). PGE2 levels in basal-like cell-CM before (basal-like cell-CM only) and after addition to fibroblasts (basal-like cell-CM on fibroblasts) were not significantly different (Figure 6A), indicating that PGE2 is produced by basal-like cells, but not fibroblasts under the cell culture conditions described here.

When indomethacin (1 or 10 μM) was added to basal-like cells, PGE2 secretion was completely abolished (Figure 6B) and basal-like cell−/+indomethacin-CM failed to upregulate HGF secretion in IPF fibroblasts (n = 4) (Figure 6C).

The PGE2 receptor antagonists, AH6809 and PF04418948, were used to inhibit the action of PGE2 on IPF fibroblasts (n = 4). PF04418948 alone (1 or 10 μM) had no significant effects on HGF secretion when compared to fibroblasts incubated with the control medium (Figure 6D). Basal-like cell-CM significantly upregulated HGF secretion, which was dose-dependently reversed by PF04418948 at 1 and 10 μM (Figure 6D). Similarly, AH6809 alone (20 μM) had no effect on HGF secretion when compared to the medium control, but significantly reversed basal-like cell-CM-induced HGF secretion by IPF fibroblasts (Figure 6E).

### Basal-Like Cell-CM Improves Lung Fibrosis *In Vivo* by a PGE2-Dependent Mechanism

**FIGURE 7:**
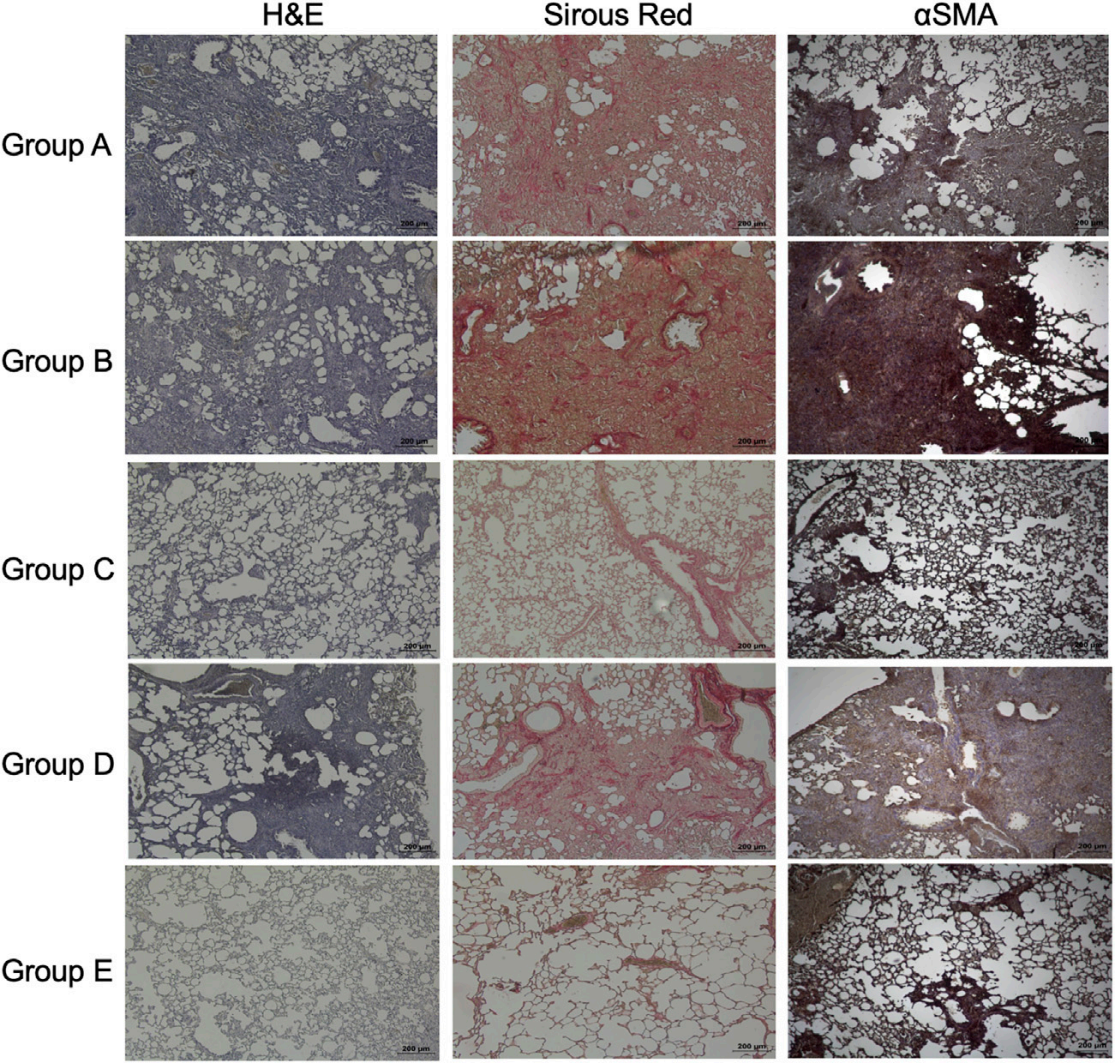
Effects of basal-like cell-CM on lung structure, collagen, or α-SMA in the bleomycin-injured rat lung. H&E staining, Sirus red staining to visualize collagen, or immunohistochemistry of α-SMA in the bleomycin-injured rat lung in group (A–E). All images were taken at day 14 (scale bar 200 µm) (A). Group legends: A, control medium; B, fibroblast-CM; C, basal-like cell-CM; D, basal-like cell-CM + PGE2 inhibitor; and E, basal-like cell-CM+PGE2 isotype control (n = 5; error bars: mean ± SEM; *p = 0.05, and ***p = 0.001).

The lung architecture was improved in the basal-like cell-CM-treated animals (group C), compared to the control groups (group A and B) as shown by H&E staining at day 7 (Figure 7). When a PGE2 antibody was added to basal-like cell-CM (group D), basal-like cell-CM did not improve lung architecture, whereas the addition of the isotype control (group E) did not alter the effect of basal-like cell-CM on lung architecture as shown by H&E staining (Figure 7). Pico Sirius staining further showed dense red-stained areas in the interstitium of the bleomycin control lung (group A) or in the lung of animals treated with F-CM (group B) (Figure 7). The group of animals treated with basal-like cell-CM (group C) did not show dense red collagen areas (Figure 7). However, blocking of PGE2 led to increased dense red collagen areas (group D), whereas the isotype control (group E) had no effect (Figure 7). Immunohistochemistry revealed dense areas of α-SMA positivity in control groups (group A and B, Figure 7). However, α-SMA-positive cells in the interstitial space in the group treated with basal-like cell-CM (group C) were not observed. Blocking of PGE2 in the basal-like cell-CM led to an increase in α-SMA-positive areas (group D), whereas the isotype control (group E) had no effect on α-SMA expression (Figure 7).

**FIGURE 8:**
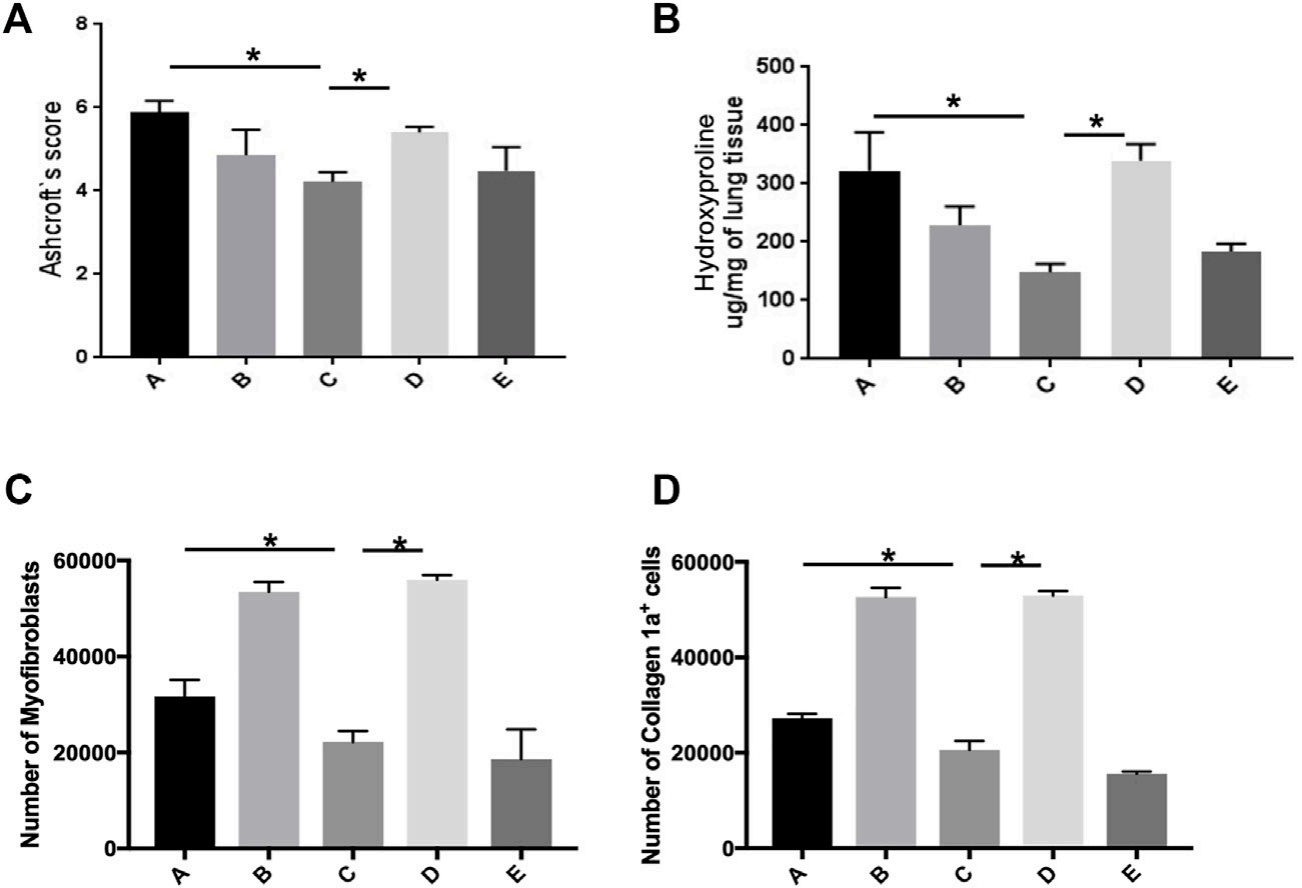
Basal-like cell-CM reduces the Ashcroft score, collagen level, and number of myofibroblasts in bleomycin-injured rat lung. Assessment of lung fibrosis using the Ashcroft scores for grading pulmonary fibrosis 14 days after bleomycin instillation **(A)**. Collagen level as measured by hydroxyproline assay at day 14. (*p ˂ 0.05) **(B)**. Number of myofibroblasts (α-SMA^+^/vimentin^+^/desmin^−^). Stopping gate was set at 30,000 recorded live events at the single cell gate. Dead and doublet cells were excluded from the analysis **(C)**. Number of α-SMA-positive cells producing collagen-1a **(D)**. A total of six groups were used (five rats per group). Group legends: A, control medium; B, fibroblast-CM; C, basal-like cell-CM; D, basal-like cell-CM + PGE2 inhibitor; and E, basal-like cell-CM + PGE2 isotype control (n = 5; error bars: mean ± SEM; *p = 0.05, and ***p = 0.001).

The Ashcroft score in fibrotic lungs treated with basal-like cell-CM (group C) was significantly reduced compared to the animals treated with the control medium (group A, Figure 8A). Application of F-CM (group B) to bleomycin-treated rats had no significant effect on the Ashcroft score (Figure 8A), when compared to group A. In presence of a PGE2 antibody in basal-like cell-CM (group D), its inhibitory effect was significantly reversed, whereas the addition of isotype control (group E) had no effect on the Ashcroft score (Figure 8A).

Collagen level of the lung in the animals treated with basal-like cell-CM (group C, 148.2 ± 14.36 µg/mg) was significantly reduced as measured by hydroxyproline assay compared to group A (332.4 ± 65.93 µg/mg) and group B (229.1 ± 31.88 µg/mg) (Figure 8B). The addition of F-CM (group B) did not significantly reduce collagen level when compared to animals treated with a control medium (group A, Figure 8B). The addition of a PGE2 antibody to basal-like cell-CM (group D) significantly reversed the inhibitory effect of basal-like cell-CM (group C) on collagen level, whereas the addition of isotype control (group E; 183.8 ± 13.33 µg/mg) had no effect (Figure 8B).

Flow cytometry measurements reveal that bleomycin-treated rats that received basal-like cell-CM (group C) have significantly lower numbers of myofibroblasts (22,232 ± 2,260 cells) compared to 31,741 ± 3,451 cells in control group A and 53,475.63 ± 2054 cells in group B. Furthermore, after PGE2 inhibition, the number of myofibroblasts increased (55,993 ± 975 cells) (group D). The cell number in group E was 6,163 ± 1,089 cells (Figure 8C).

Similarly, collagen-1a-expressing α-SMA cells in animals treated with basal-like cell-CM (group C) were reduced to 20,605 ± 1,846 cells compared to animals that received F-CM (group B) (52,643 ± 1,955 cells) and control animals (group A) (27,237 ± 942 cells). Furthermore, after PGE2 inhibition, the number of cells increased, 52,913 ± 955 (group D). The cell number in group E was 15,640 ± 444 cells (Figure 8D).

## Discussion

In this study, we confirmed the presence of KRT5+/KRT17+ basal-like cells in the peripheral lung of IPF patients, localized mainly within areas of pathological tissue remodeling with bronchiolization and honeycomb cysts. We cultured these basal-like cells *in vitro* and investigated the effects of their conditioned medium (CM) on IPF fibroblasts *in vitro* and in a rat model of pulmonary fibrosis *in vivo*. We showed that basal-like cell-CM inhibits the expression and deposition of Col1A1 and upregulates MMP-1 RNA and protein expression, without having effects on TIMP-1 in IPF fibroblasts. The secretion of anti-fibrotic HGF was enhanced, and α-SMA expression was reduced in IPF fibroblasts by basal-like cell-CM. In a rat model of bleomycin-induced lung fibrosis, intratracheal instillation of basal-like cell-CM significantly reduced collagen level and α-SMA expression and improved lung structure, thus supporting the *in vitro* findings. Furthermore, PGE2 was identified as a crucial factor mediating the effects of lung-resident basal-like cell-CM. Our data suggest that basal-like cells secrete factors that limit aberrant fibroblast activation and their differentiation into myofibroblasts.

In line with our findings, a number of previous studies showed the disease-enriched presence of airway basal-like cells within the peripheral lung (Chilosi et al., 2002; Smirnova et al., 2016; Prasse et al., 2019; Fernanda de Mello Costa et al., 2020). Furthermore, a small population of aberrant basaloid cells, expressing some, but not all, canonical basal cell markers (KRT5-/KRT17+) was recently identified in peripheral lung tissue of IPF patients (Adams et al., 2020; Habermann et al., 2020) The role and function of these cells in IPF pathogenesis remains, however, largely unknown. The presence of basal-like cells in bronchoalveolar lavage (BAL) fluid or in the alveolar region of lung tissue derived from the IPF patients was associated with increased mortality (Prasse et al., 2019), pathological bronchiolization, and honeycomb formation (Chilosi et al., 2002; Prasse et al., 2019). On the contrary, findings from studies in mice suggested that basal-like cells regenerate the alveolar epithelium by their differentiation into AT2 cells (Kumar et al., 2011; Vaughan et al., 2015; Zuo et al., 2015; Yuan et al., 2019). However, there is growing evidence that the cells’ capacity to regenerate AT2 cells is limited (Fernanda de Mello Costa et al., 2020).

The cross-talk between alveolar epithelial cells and adjacent fibroblasts seems crucial for maintaining normal lung homeostasis, and a loss of AT2 cell-secreted anti-fibrotic mediators may result in uncontrolled fibroblast differentiation and collagen secretion (Bagnato and Harari 2015; Knudsen et al., 2017). Since basal-like cells are present in the alveolar space of the fibrotic lung, we here determined the effects of their conditioned medium on IPF lung fibroblasts and in bleomycin-challenged rats. Surprisingly, we observed a number of anti-fibrotic effects mediated by basal-like cell-CM in our *in vitro* and *in vivo* models.

Prostaglandin E2 (PGE2), a bioactive eicosanoid that regulates many biologically important processes (Bozyk and Moore 2011), was identified as a crucial secretory factor mediating the effects in basal-like cell-CM. Similar to the basal-like cell-CM, recombinant PGE2 dose-dependently reduced Col1A1 and increased HGF in IPF fibroblasts. Furthermore, blocking PGE2 in basal-like cell-CM reversed HGF upregulation in IPF fibroblasts *in vitro* and total collagen and α-SMA-expressing cells in bleomycin-injured rat lung. In line with our data, other studies showed that PGE_2_ mediates antifibrotic effects, primarily by inhibiting fibroblast proliferation, migration, collagen synthesis, and moreover, limiting lung myofibroblast differentiation (Bozyk and Moore 2011; Epa et al., 2015; Mukherjee et al., 2019). However, in a mixture of different mediators as in a conditioned medium, it seems difficult to appoint all effects to a single mediator, which is supported by our data showing that recombinant PGE2 failed to upregulate MMP-1. Furthermore, Col1A1 expression was reduced not only by recombinant PGE2 but also by several other recombinant cytokines.

In contrast to our findings, a recent pre-print reported enhanced collagen and α-SMA in lung fibroblasts treated with airway basal cell-derived CM (Jaeger et al., 2020). However, these putative contrasting findings likely arise from the use of different sampling locations. Basal cells reported in the pre-print were obtained by bronchial brushings, while we cultured the cells from peripheral lung tissue. Importantly, this may indicate that basal cells display differential characteristics and functional properties depending on their location within the lung.

It is important to mention that basal cells in the human lung represent a heterogenous cell population including multipotent, proliferating, or differentiating basal cells (Carraro et al., 2020; Travaglini et al., 2020), as well as the recently described fibrosis-enriched KRT5-/KRT17 + basaloid cells (Adams et al., 2020; Habermann et al., 2020). Cultured basal-like cells used in our experiments unlikely represent the newly described aberrant basaloid cells as they, in contrast to aberrant basaloid cells (Adams et al., 2020; Habermann et al., 2020), express high levels of KRT5. Furthermore, due to the low number of aberrant basaloid cells within fibrotic tissue and their specific location on the surface of fibroblastic foci (Adams et al., 2020; Habermann et al., 2020), the culture method described here seems insufficient to culture such a rare cell population. scRNA-seq. analysis demonstrated the presence of basal, differentiating basal, and proliferating basal cells within the cultured cell population. Furthermore, some of the cells showed transcriptional similarities to secretory or ciliated epithelial cells, suggesting spontaneous cell differentiation under the culture conditions described here. We cannot exclude that the secretome of the different basal cell subsets may differ and they, therefore, may contribute distinctly to the CM effects reported here. The use of an epithelial cell-specific culture medium would likely inhibit spontaneous cell differentiation, but would also limit the use of basal-like conditioned medium on cultured fibroblasts as these cells cannot be cultured in an epithelial cell-specific medium. It is also to consider that the *in vitro* culture of the cells may alter the composition of their secretome and, therefore, not fully reflect the cells’ secretome and its effects *in vivo;* importantly, the cells’ interaction with their microenvironment is not represented in our *in vitro* model*.* Furthermore, apart from their paracrine effects, other functional properties such as the cells’ differentiation capacity may contribute to their pro- or anti-fibrotic properties. Further studies are, therefore, needed in order to get a full understanding of the cells’ functional role in IPF. Furthermore, a seemingly missing validation cohort of basal-like cells grown from non-fibrotic tissue might be a point of criticism. However, we earlier showed that, under standardized cell culture conditions, basal-like cells readily grew from peripheral fibrotic, but only rarely from non-fibrotic tissue (Hostettler et al., 2017), making it difficult to provide a validation cohort of cultured non-fibrotic basal-like cells. Moreover, as a proof-of-concept study, we have used the bleomycin animal model. Although it is the most commonly used preclinical model, it has limitations of not being able to fully represent the human pathology.

Taken together, basal-like cells seem to re-epithelialize the damaged alveolar epithelium to maintain barrier function after severe lung injury. We here show that basal-like cells secrete factors that limit aberrant fibroblast activation. However, despite these beneficial effects, previous data indicate a potential harmful role of basal cells in the pathogenesis of lung fibrosis (Prasse et al., 2019). This discrepancy might be explained by the cells’ limited capacity to differentiate into functional AT2 cells, which might hinder normal regeneration of the alveolar epithelium in the long term. Therefore, studying the capacity of basal-like cells to differentiate into functional AT2 cells and how this may be influenced by their specific microenvironment is of utmost interest.

## Data Availability

The datasets presented in this study can be found in online repositories. The names of the repository/repositories and accession number(s) can be found in the following: https://www.ncbi.nlm.nih.gov/geo/, GSE145439.
