# Supplementary figures and images for: Basal-Like Cell-Conditioned Medium Exerts Anti-Fibrotic Effects In Vitro and In Vivo
Source: Front Bioeng Biotechnol. 2022 Mar 8;10:844119. doi: 10.3389/fbioe.2022.844119 (PMC8957873; doi:10.3389/fbioe.2022.844119)

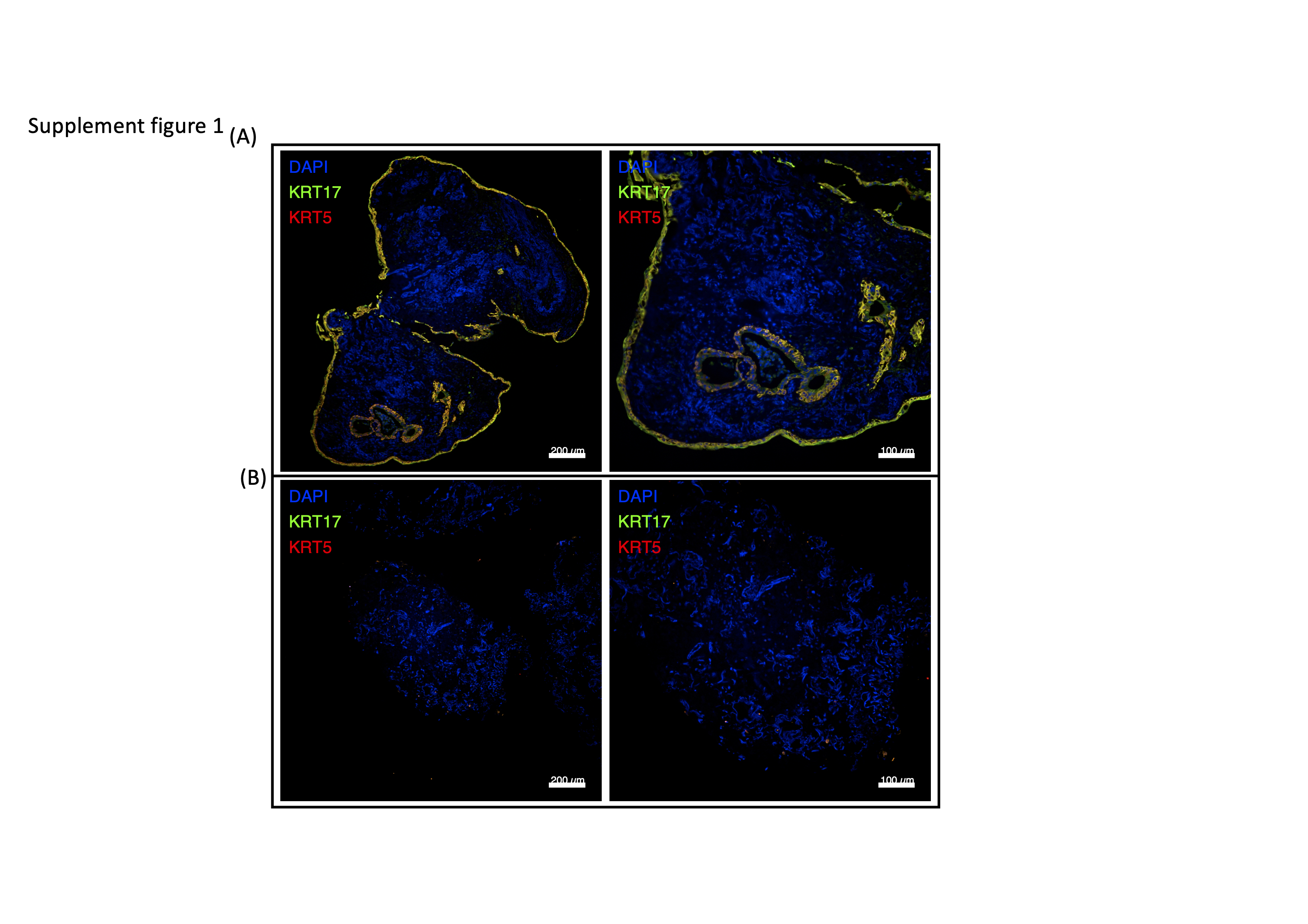

Supplement: Supplementary file 2 [file Image1.TIFF]

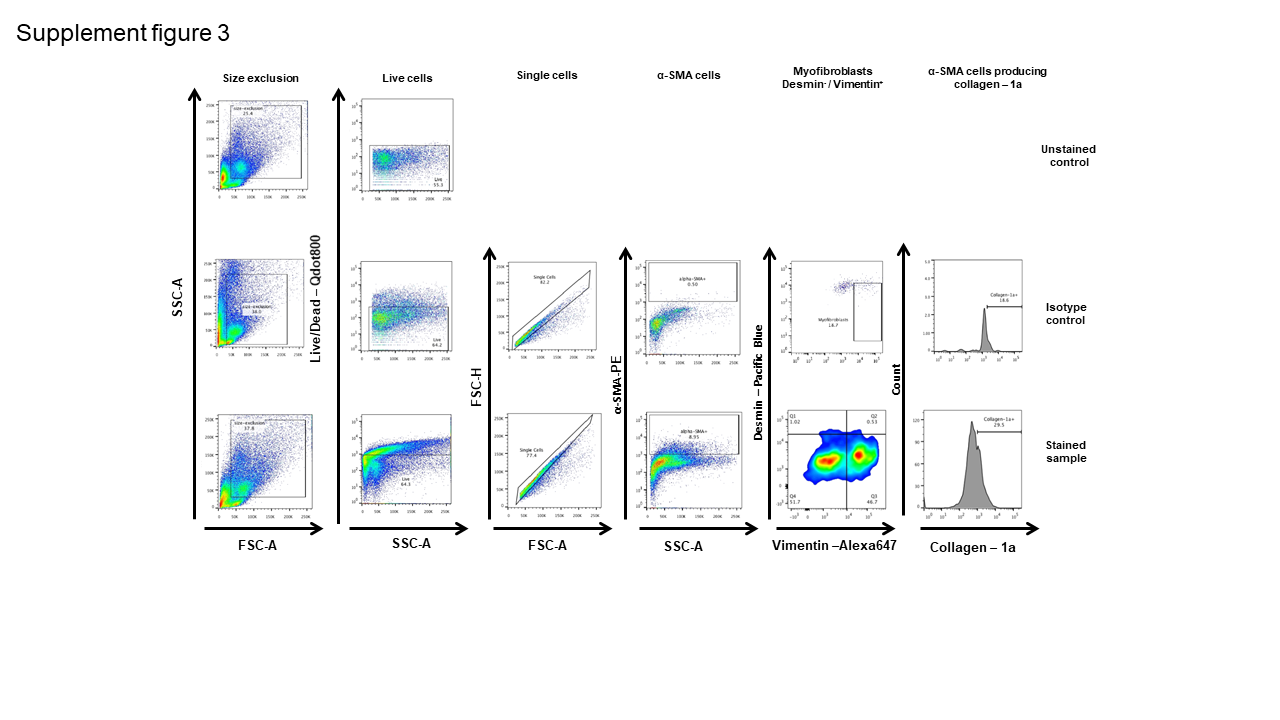

Supplement: Supplementary file 3 [file Image3.TIF]

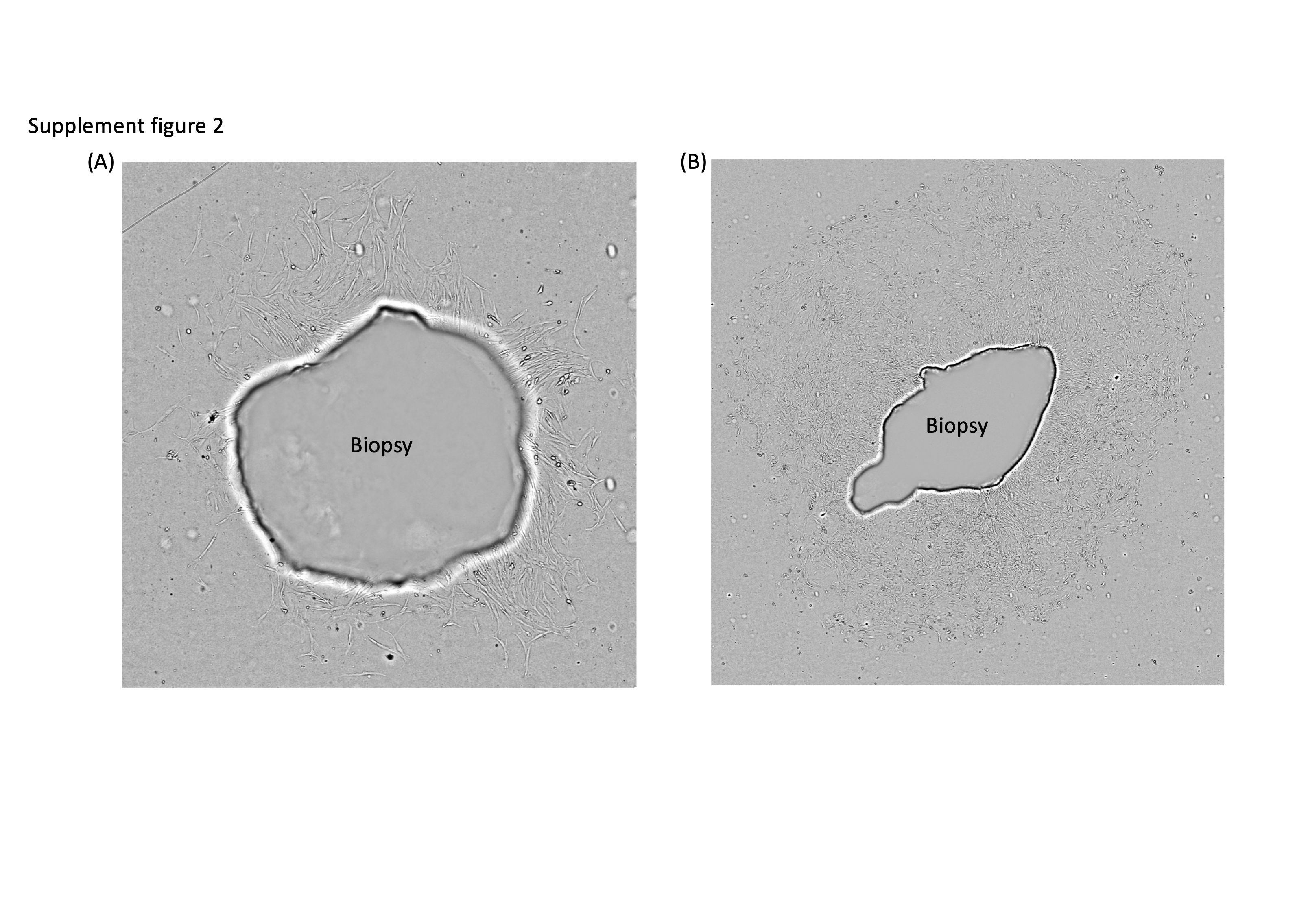

Supplement: Supplementary file 5 [file Image2.TIFF]
