# Supplementary material for: Basal-Like Cell-Conditioned Medium Exerts Anti-Fibrotic Effects In Vitro and In Vivo
Source: Front Bioeng Biotechnol. 2022 Mar 8;10:844119. doi: 10.3389/fbioe.2022.844119 (PMC8957873; doi:10.3389/fbioe.2022.844119)
Supplement: Supplementary file 6 [file DataSheet1.DOCX]

Supplementary Material

### **Supplementary materials and methods**

### Single cell RNA-sequencing

Single-cell suspensions were generated from basal-like cells grown from cultured peripheral lung tissue biopsies from 3 IPF, controlled for viability, and loaded on separate wells of a 10X Genomics Chromium Single Cell Controller in 2 batches (IPF1 on April 8^th^ 2019, and IPF2 and 3 on May 27^th^ 2019) for an expected recovery of 10,000 cells.

Single cell capture and cDNA and library preparation were performed at the Genomics Facility Basel of the ETH Zurich, Basel with a Single Cell 3’ v3 Reagent Kit (10X Genomics) according to the manufacturer’s instructions. Sequencing was performed on one flow-cell of the Illumina NextSeq 500 platform (with 56nt-long R2 reads). Read quality was assessed with the FastQC tool (version 0.11.5). Sequencing files were processed with Kallisto (version 0.46.0) and BUStools (version 0.39.2) to perform sample and cell demultiplexing, and pseudo-alignment of reads to human transcripts (Ensembl release 96)(Melsted et al., 2019; Melsted et al., 2019). Processing of the UMI counts matrix was performed using the Bioconductor packages DropletUtils (version 1.5.4)(Griffiths et al., 2018; Lun et al., 2019), scran (version 1.12)(Lun et al., 2016; Vallejos et al., 2017) and scater (version 1.12)(McCarthy et al., 2017), following mostly the steps illustrated in the OSCA book (http://bioconductor.org/books/release/OSCA/)(Amezquita et al., 2019; Lun et al., 2016). After quality filtering, the resulting dataset consisted of 17,869 genes and 7,498 cells derived from patient 1, 905 cells from patient 2, and 550 cells from patient 3. Systematic differences between batches were removed using the Seurat integration pipeline (Stuart et al., 2019) after renormalizing the dataset using SCTransform (Hafemeister and Satija 2019). The 2D *t*-distributed stochastic neighbor embedding (tSNE) used for visualization of cells was calculated using the principal components of the integrated dataset as input and a perplexity of 100. For the annotation of cell-types, a reference scRNA-seq atlas of cell types in the human lung (Travaglini et al., 2020) was used. The UMI count matrix and annotation of cells were downloaded from <https://www.synapse.org/#!Synapse:syn21041850/files/>. A “pseudo-bulk” approach (Lun and Marioni 2017) was then used, whereby UMIs from lung samples cells from each cell type and donor were aggregated (if at least 30 cells were available for a cell type and donor pair). The resulting log-counts per million UMIs (logCPM) matrix for the 165 pseudo-bulk samples was used for annotation using The Bioconductor package SingleR (version 1.2.4) (Aran et al., 2019). For simplicity in the resulting annotation, the different subsets of immune cells were grouped, as well as the fibroblast/myofibroblast/smooth muscle subsets. We also ignored the proximal, medial or distal lung site of sampling.

*Human lung tissue staining*

Peripheral human lung tissue was fixed with 4% paraformaldehyde and embedded in paraffin. The paraffin blocks were cut (7µm) and then rehydrated using Ultraclear-ethanol series for deparaffinization. For heat-induced antigen retrieval, all sections were boiled at 95°C for 30min in a citrate-based antigen unmasking solution. Tissue slides were blocked with 0.5% Triton X-100, 3% bovine serum albumin (BSA) and incubated with antibodies (Supplement table 1). Slides were mounted with ProLong Gold antifade reagent, imaged on a Nikon Ti2 fluorescence microscope and then analyzed by ImageJ. In some selected cases, lung tissue was used for cell culture before fixation for staining; tissue was first cut in small pieces, placed in culture till cell growth was observed and was subsequently fixed and embedded in paraffin.

*Immunohistochemistry*

For immunohistochemistry the slides were pretreated in sodium citrate buffer (100 mM, pH 7.0) and boiled in a microwave oven for 5 mins. Slides were washed three times with Tris Buffer Saline with 0.1% Tween-20 (TBST). Thereafter, tissue slides were incubated with primary antibody α-SMA 1:100 overnight at 4°C. Slides were further processed using Polink HRP immunohistochemistry kit following the manufacturer’s instructions. The slides were then counterstained using hematoxylin, and mounted. Images were acquired using Leica DM4000D

*Histology and Sirius red staining*

Routine Hematoxylin and Eosin (H&E) staining and Sirus red staining was performed with formalin-fixed tissue sections. Histological grading of fibrosis was done by a trained pathologist blinded to the study following the scoring system of Ashcroft (Ashcroft et al., 1988). For visualization of collagen Sirus red staining was applied. Briefly, after dewaxing and hydrating the paraffin sections were kept in hematoxyline for nuclear stain. After washing in running water the slides were incubated with picro-sirius red for 1 hour and washed with acidified water, dehydrated cleared with xylene and mounted. Images were acquired using Leica DM4000D.

*Hydroxyproline Assay*

Lungs were analysed for collagen content as initially described by Woessner (Woessner 1961). The lungs were snap frozen after having measured the weight. The frozen lungs where homogenized in 1x PBS and 1 mL of the homogenate was treated with 10% trichloroactic acid (TCA), hydrolyzed with 6 M hydrochloric acid (18 hours at 110°C). Oxidation was initiated by 1 ml of chloramin T-reagent for 20 minutes at room temperature and stopped by addition of 1 ml of 3.15 M perchloric acid. After incubation with Ehrlich reagent (p-dimethylaminobenzaldehyde) for 20 minutes at 55–65°C the absorbance of each sample was measured at 557 nm. A standard curve was generated using known concentrations of reagent grade hydroxyproline, as described before (Gazdhar et al., 2013; Tamo et al., 2018).

*Flow cytometry*

10^6^ freshly isolated cells were suspended in washing buffer (WB) containing PBS, 0.1% BSA and 0.09% NaN3. Cell suspensions were washed with PBS, stained, for viability with the LIVE/DEADTM staining kit, according to the protocol provided. For further stainings, cells were fixed and permeabilized with Cytofix/Cytoperm and were stained with α-SMA-PE, Vimentin-Alexa 647, Desmin conjugated with: Goat-anti-Rabbit IgG Pacific Blue and Collagen-1a-Alexa 488 at dilutions as shown on Table 2. Cells were incubated on ice for 30min, protected from light and were washed three times with washing buffer and analysed using BD LSRII instrument.

**Supplement Figure 1:** Small pieces of peripheral lung tissue were cultured in DMEM/10%FCS for up to 7 days. As soon as cell growth was observed, five pieces showing fibroblast- and five pieces showing basal-like cells outgrowth for each patient (n=5) were fixed in 4% paraformaldehyde and embedded in paraffin. KRT17+/KRT5+ basal-like cells were only present in tissue pieces showing outgrowth of basal-like cells (A), but were absent in tissue pieces showing fibroblast outgrowth (B).

**Supplement Figure 2:** Phase contrast pictures showing the distinct morphology of fibroblasts (A) or basal-like cells (B) growing from peripheral lung tissue pieces cultured in DMEM/10%FCS.

**Supplement Figure 3:** Flow cytometry gating strategy. Representative gating strategy is shown. After size and doublet exclusion, dead cell exclusion is shown. On the upper panel, unstained samples were used to set the gate for the live cells. On the middle panel, gating strategy based on isotype controls is shown and on the lower panel, the expression of α-SMA, vimentin, desmin and collagen-1a markers are shown. This gating strategy was applied to all samples (n=5 animals/group).

Amezquita, R. A., Lun, A. T. L., Becht, E., Carey, V. J., Carpp, L. N., Geistlinger, L., et al. (2019). Orchestrating single-cell analysis with Bioconductor. Nat Methods.

Aran, D., Looney, A. P., Liu, L., Wu, E., Fong, V., Hsu, A., et al. (2019). Reference-based analysis of lung single-cell sequencing reveals a transitional profibrotic macrophage. Nat Immunol; 20: 163-172.

Ashcroft, T., Simpson, J. M. and Timbrell, V. (1988). Simple method of estimating severity of pulmonary fibrosis on a numerical scale. J Clin Pathol; 41: 467-470.

Gazdhar, A., Temuri, A., Knudsen, L., Gugger, M., Schmid, R. A., Ochs, M., et al. (2013). Targeted gene transfer of hepatocyte growth factor to alveolar type II epithelial cells reduces lung fibrosis in rats. Hum Gene Ther; 24: 105-116.

Griffiths, J. A., Richard, A. C., Bach, K., Lun, A. T. L. and Marioni, J. C. (2018). Detection and removal of barcode swapping in single-cell RNA-seq data. Nat Commun; 9: 2667.

Hafemeister, C. and Satija, R. (2019). Normalization and variance stabilization of single-cell RNA-seq data using regularized negative binomial regression. Genome Biology; 20: 296.

Lun, A., McCarthy, D. and Marioni, J. (2016). A step-by-step workflow for low-level analysis of single-cell RNA-seq data with Bioconductor [version 2; referees: 3 approved, 2 approved with reservations]. F1000Research; 5.

Lun, A. T., Bach, K. and Marioni, J. C. (2016). Pooling across cells to normalize single-cell RNA sequencing data with many zero counts. Genome Biol; 17: 75.

Lun, A. T. and Marioni, J. C. (2017). Overcoming confounding plate effects in differential expression analyses of single-cell RNA-seq data. Biostatistics.

Lun, A. T. L., Riesenfeld, S., Andrews, T., Dao, T. P., Gomes, T., participants in the 1st Human Cell Atlas, J., et al. (2019). EmptyDrops: distinguishing cells from empty droplets in droplet-based single-cell RNA sequencing data. Genome Biol; 20: 63.

McCarthy, D. J., Campbell, K. R., Wills, Q. F. and Lun, A. T. L. (2017). Scater: pre-processing, quality control, normalization and visualization of single-cell RNA-seq data in R. Bioinformatics; 33: 1179-1186.

Melsted, P., Booeshaghi, A. S., Gao, F., Beltrame, E., Lu, L., Hjorleifsson, K. E., et al. (2019). Modular and efficient pre-processing of single-cell RNA-seq. bioRxiv: 673285.

Melsted, P., Ntranos, V. and Pachter, L. (2019). The barcode, UMI, set format and BUStools. Bioinformatics; 35: 4472-4473.

Stuart, T., Butler, A., Hoffman, P., Hafemeister, C., Papalexi, E., Mauck, W. M., 3rd, et al. (2019). Comprehensive Integration of Single-Cell Data. Cell; 177: 1888-1902 e1821.

Tamo, L., Simillion, C., Hibaoui, Y., Feki, A., Gugger, M., Prasse, A., et al. (2018). Gene Network Analysis of Interstitial Macrophages After Treatment with Induced Pluripotent Stem Cells Secretome (iPSC-cm) in the Bleomycin Injured Rat Lung. Stem Cell Rev; 14: 412-424.

Travaglini, K. J., Nabhan, A. N., Penland, L., Sinha, R., Gillich, A., Sit, R. V., et al. (2020). A molecular cell atlas of the human lung from single-cell RNA sequencing. Nature; 587: 619-625.

Vallejos, C. A., Risso, D., Scialdone, A., Dudoit, S. and Marioni, J. C. (2017). Normalizing single-cell RNA sequencing data: challenges and opportunities. Nat Meth; 14: 565-571.

Woessner, J. F. (1961). The determination of hydroxyproline in tissue and protein samples containing small proportions of this imino acid. Archives of Biochemistry and Biophysics; 93: 440-447.
